# Internet of Things: The Optimal Generation Rates under Preemption Strategy in a Multi-Source Queuing System

**DOI:** 10.3390/e23081055

**Published:** 2021-08-16

**Authors:** Tianci Zhang, Shutong Chen, Zhengchuan Chen

**Affiliations:** 1 School of Microelectronics and Communication Engineering, Chongqing University, Chongqing 400044, China; 20183875@cqu.edu.cn (T.Z.); 20183753@cqu.edu.cn (S.C.); 2 National Mobile Communications Research Laboratory, Southeast University, Nanjing 210000, China

**Keywords:** Internet of Things (IoT), information freshness, age of information (AoI), multi-source M/M/1/1 preemptive queuing model

## Abstract

With the rapid development and wide application of the Internet of Things (IoT), how to provide timely and fresh information for strategic analysis and decision-making has become a key issue. Recent studies have shown that preemption strategies are of great importance to the improvement of information freshness. In view of this, we focus on the multi-source preemptive queuing model and investigate how to control the generation rate of each source to achieve the optimal overall information freshness. Specifically, we consider two typical preemption strategies: self-preemption strategy and global-preemption strategy. Noting that the urgency requirements of the systems on the data of each source are different, we propose the weighted average age of information (AoI) to characterize the overall information freshness of the system. For the self-preemption strategy, we prove that the optimal generation rate allocation is a convex problem and present an efficient algorithm to find the optimal solution. Additionally, we also derive a closed-form approximate optimal solution under light load cases to meet the demands for rapid deployment. For the global-preemption strategy, we directly derive the closed-form optimal solution of the corresponding problem. By comparing the optimized weighted average AoIs, the performance achieved by the global-preemption system was better than that achieved by the self-preemption system in terms of the overall timeliness. The numerical analysis verified the correctness of the theoretical analysis and that the proposed approximate solution had high accuracy not only under light load cases but also under other cases.

## 1. Introduction

The Internet of Things (IoT) is yet another revolutionary development in the information technology industry. Currently, the IoT is an important productivity contribution to the rapid development of the world, integrating various information sensing devices with the Internet to form a huge network that achieves sensing and real-time control of the real world in time and space [1]. By using the technology of the IoT, information sensing devices, such as radio frequency identification, infrared sensors, and laser scanners, can collect a wide variety of information used for analysis and decision-making of upper level data-driven applications.

IoT has been widely used in various fields, such as Intelligent Transport System (ITS), Smart Grid (SG), and Internet of Vehicles (IoV) [2,3,4]. It is worth noting that, for a considerable part of practical applications, the freshness of the data streams and status updates in the IoT are of great significance for strategies analysis and decision-making [5]. For the autonomous driving system, for example, if it receives obsolete information at the receiver, it will cause an obvious reduction in the accuracy and reliability of the system decision and may even cause serious accidents.

In actual application scenarios, status updates from different sources correspond to different information and the data processing equipments may have different urgency requirements for these status updates. For example, in the UAV networks, the control terminal hopes to receive the UAV’s flight speed, acceleration, and position updates with high real-time performance. However, the timeliness requirements for information, such as the engine temperature and remaining fuel, are relatively low.

Based on the above backgrounds, in this work, we focus on optimizing the overall timeliness of a multi-source state update system, in which the urgency requirements of the receiver for update packets from different sources are different. To characterize common problems in preemptive queuing systems, we model the system as a multi-source M/M/1/1 preemptive queuing model. Specifically, we consider two typical preemption strategies: the self-preemption strategy and global-preemption strategy. In addition, we introduce the weight factor of the age of information (AoI) to represent the difference of the urgency requirements between different sources. The optimization of the overall timeliness can be achieved by finding the optimal generation rate of each source, which minimizes the weighted average AoI.

### 1.1. Contributions

The main contributions of this paper are summarized as follows.

- The multi-source status update system is modeled as a multi-source M/M/1/1 queuing model with preemption. To characterize the overall timeliness of the system, we introduce the weight factor and the weighted average AoI measure. A generation rate control scheme, which varies the generation rate of different sources according to their different urgencies, is proposed to improve the overall timeliness performance. Additionally, we formulate the optimal generation rate problem as a weighted average AoI minimization problem.
- For the self-preemption strategy, we prove that the weighted average AoI minimization is a convex problem and propose a convex optimization algorithm to find the optimal generation rate. To meet the demands of rapid deployment in some scenarios, a set of approximate optimal generation rates for the system with light load is proposed. To facilitate the further analysis and comparison with the overall freshness of the self-preemptive strategy, we also derive a set of upper bound and lower bound for the minimal weighted average AoI. For the global-preemption strategy, we explicitly present a closed-form expression for the corresponding optimal generation rates. We surprisingly found that the proposed approximate optimal generation rates for the self-preemption strategy were the same as the optimal generation rates for the global-preemption strategy. In addition, the lower bound of the minimal weighted average AoI of the self-preemption strategy was the same as that of the global-preemption strategy, manifesting that the performance achieved by the global-preemption system was better than that achieved by the self-preemption system in terms of the overall timeliness.
- Numerical analysis verified our theoretical results. In particular, we show that optimizing the generation rate significantly improved the overall timeliness performance of the system. According to the obtained relative error between the true optimum and approximation, the proposed approximate optimal generation rates for the self-preemption strategy approached the true optimum well in the light load case and even in other cases, which provides great application value for the proposal of the approximate optimal generation rate.

### 1.2. Organization

The rest of this paper is structured as follows: the related works are presented in Section 2. In Section 3, we present the system model and formulate the optimization problems. In Section 4, we investigate the optimal status update generation rate for both the self-preemption strategy and global-preemption strategy. Numerical simulations that match our propositions are presented in Section 5. Finally, our work is concluded in Section 6.

## 2. Related Works

In this Section, we present the related works to give broader perspective and further understandings about our work.

In 2011, AoI was proposed to effectively characterize information freshness [6]. AoI at any time in the system is defined as the time elapsed since the generation of the latest accepted state update. Thus, AoI perfectly presents the freshness of the latest information received by the receiver. However, traditional timeliness metrics, such as packet delay, only focus on the time elapsed by each packet in the system, and cannot evaluate the information freshness of the receiver as AoI can [7].

Based on single-source M/M/1 (We follow the Kendall notations for identifying the statistics of different queues.), M/D/1, D/M/1, and M/G/1 First-Come-First-Served (FCFS) queuing models, the average AoI has been derived in [6,8]. The work in [8] obtained several formulas for the stationary distributions of the AoI and the peak AoI in the FCFS G/G/1 queue. Furthermore, the authors of [9] derived a general expression for the stationary distribution of the AoI in the single-source queuing model. The research of [10] was the first work to investigate the average AoI in a multi-source setup, which derived the average AoI for a multi-source FCFS M/M/1 queueing model. The authors of [11] derived a closed-form expression for the average AoI of each source in the multi-source M/G/1 FCFS queuing model.

Package management policies are often considered as an effective tool to improve the freshness of state updates, e.g., related works were carried out in [9]. According to the definition of AoI, shortening the buffer size of the queue is considered as an intuitive policy, in which the packets that arrive while the server is busy are discarded to avoid receiving outdated packets. In [12,13], it was shown that a large buffer size does not always improve the information freshness.

On the other hand, researchers have also focused on preemption systems. Essentially, the preemption strategies prevent the accumulation and the sojourn of the status update packets in buffer such that the newly arrived status update packets can be directly served. Therefore, generally, the preemption strategies can ensure that the information received is as fresh as possible except for some special cases, e.g., [14], where the service time follows Gamma distribution. The work [15] derived the average AoI of the single-source M/G/1/1 preemptive queuing model in a closed form.

For the queuing model with single source Poisson arrival, the authors of [14] calculated the average AoI and the average peak AoI under two different schemes: Last-Come-First-Served (LSCF) with preemption and LCFS without preemption, respectively, where the service time follows a Gamma distribution. For the multi-source M/G/1/1 preemptive queuing model, the commonly used preemption strategies are divided into two types: self-preemption strategies and global preemption strategies. (Global-preemption means that any stream can preempt itself and any other stream. However, in the self-preemption strategy, any stream can only preempt itself).

Based on the multi-source M/G/1/1 queuing model with global-preemption, the authors of [16] derived a closed-form expression for the average AoI and the average peak AoI of each stream. After that, in the work [17], a closed-form expression for the average AoI of each source in the multi-source M/M/1/1 queuing model with self-preemption was derived. The authors of [17] also compared the self-preemption strategy to the global-preemption and found that the former performed better in the multi-source M/M/1/1 queue.

There have been some optimization works based on AoI. In [18], the authors investigated the minimization of the AoI of the date collected by the ground sensor nodes in the UAV-assisted wireless powered IoT system. The work [19] introduced a deep reinforcement learning-based approach to minimize the AoI in the networks whose topology was without prior assumptions. A recent work [20] designed an optimal offline solution and an effective online solution to minimize the average AoI in energy harvesting-based networked embedded systems with energy constraints.

The authors of [21] investigated the distribution of AoI in a wireless networked control system with two hops—based on which, the authors minimized the tail of the AoI distribution respect to the frequency of generating information updates, i.e., the generation rate of a source. The authors utilized a probabilistic scheduling method to minimize the AoI in transmissions with truncated channel inversion while satisfying an average power constraint in [22].

Furthermore, in a multi-source system, optimizing the generation rate of the state updates of each source is an important scheme to improve the overall timeliness of the system. For instance, the study in [16] showed that setting equal generation rates for all sources achieved the lowest total average AoI and total average peak AoI of all sources for the multi-source M/G/1/1 queuing model with global-preemption.

In [13], the peak average AoI was used to characterize the information freshness of multi-source M/G/1 and multi-source M/G/1/1 queueing systems. The seeking of the optimal generation rate of each source was formulated by using quasi-convex optimization, and the structural characteristics of the optimal solution were obtained. Additionally, the investigation of [23] showed that the optimal update rate significantly reduced the AoI violation probability for a wide range of AoI constraints.

## 3. System Model and Problem Formulations

In this section, we model the multi-source preemptive queuing system in IoT as a common multi-source M/M/1/1 preemptive queuing system and introduce the weighted average AoI to characterize the overall timeliness of the system. Additionally, we consider two common preemption strategies—the self-preemption strategy and global-preemption strategy—and then formulate optimization problems w. r. t. the generation rate of each source under these two strategies.

### 3.1. System Model

**Figure 1:**
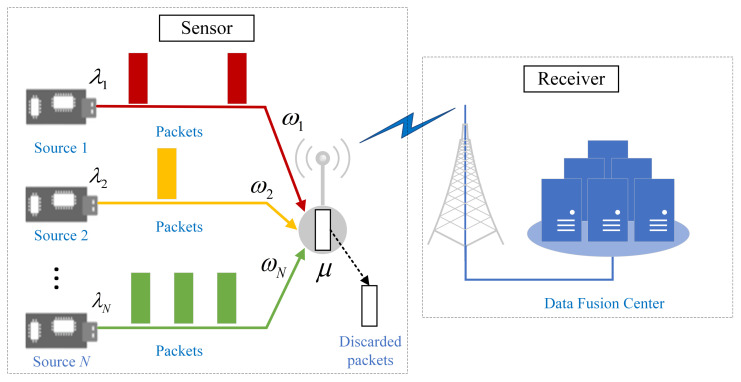
A multi-source M/M/1/1 queuing system with preemption. Source *i* generates packets with rate $\lambda_{i}$ based on the Poisson process, whose urgency requirement is characterized by $\omega_{i}$. After that, the sensor sends the packets according to the preemption strategies—the global-preemption strategy and self-preemption strategy. Successfully sent packets depart the sensor with rate μ. The date fusion center only receives the packets that are successfully sent.

In this paper, we consider a multi-source queuing system with preemption, as shown in Figure 1.

A sensor collects status updates from *N* sources and sends them to a receiver node, e.g., a data fusion center, for analyses and decision-making. For example, for an automatic driving system, it needs to send information, such as the vehicle speed and engine temperature to the remote receiving end to obtain real-time analysis and instruction generation. The sensors always send newly arrived data to improve the timeliness of the data at the receiving end, thereby, ensuring the safety of the passengers.

The status update sources are indexed by $i\in I:=\left\{1,2,\cdots ,N\right\}$. We assume that each source *i* generates status updates independently according to a Poisson process with rate $\lambda_{i}$. We denote the sum of the generation rates of all sources as the total generation rate λ, i.e., $\lambda =\sum_{i=1}^{N}\lambda_{i}$. Moreover, we assume that the arrived status updates would subsequently enter a length-1 queue with preemption, which means there is no buffer to store the incoming updates.

Taking into account the length variance of status updates, the reliability of the wireless channel, and the signal quality of the receiver, we also assume that the time used for successfully transmitting a status update to the receiver follows a negative exponential distribution with parameter μ. Therefore, we shall describe this system as a multi-source M/M/1/1 system with preemption. The server utilization of source *i* and the whole system are denoted as $\rho_{i}=\lambda_{i}/\mu$ and $\rho =\lambda /\mu =\sum_{i=1}^{N}\rho_{i}$, respectively.

*Preemption Strategies:* Two typical preemption strategies are considered for the multi-source system, i.e., self-preemption and global-preemption [17]. Specifically, taking into account that different data streams represent different attributes and connotations, and that the packet scheduling and management of the queue in each single stream can guarantee the independent statistical characteristics of each stream, we consider the self-preemption strategy, in which the packets can only preempt that of the same source but not any others.

On the other hand, since the management of packets in each data stream will increase the complexity of scheduling, we also consider a preemption strategy that does not distinguish between data streams, i.e., a global-preemption strategy, in which the packets can preempt not only that of the same source but also that of any other source.

### 3.2. Problem Formulations

In most cases, IoT-based system requires the received information to be as timely and fresh as possible. Hence, AoI, which is defined as the time elapsed since the generation of the latest successfully received information, would be a proper metric for characterizing the overall timeliness of status updates at the receiver. Generally, status updates from different sources contain different information, and the receiver has different urgency requirements for these updates. That is, different sources have different urgency.

Recall that, in the mentioned automatic driving system, a higher urgency requirement is required for vehicle speed and a lower urgency requirement is required for engine temperature. Therefore, we introduce weight factors to the average AoI of each status update source and use the weighted average AoI to quantify the overall timeliness of the system. Specifically, the weighted average AoI is defined as

(1) $$\begin{array}{c} \bar{\Delta }:=\sum_{i=1}^{N}\omega_{i}\Delta_{i}, \end{array}$$

where $\Delta_{i}$ represents the average AoI of source *i* and $\omega_{i}\in \left[0,1\right]$ is the weight factor that characterizes the urgency requirement on source *i*. The sum of the weight factors is set to be 1, i.e., $\sum_{i=1}^{N}\omega_{i}=1$. It is clear that the larger $\omega_{i}$ is, the more urgent the requirement for source *i* is.

To improve the overall timeliness performance of the system significantly, we chose the generation rate control scheme, which reasonably adjusts the generation rate of different sources under the fixed total generation rate λ. In practice, e.g., in the exemplary automatic driving system, both the bandwidth allocated for status update transmission and energy allocated for sensing at wireless connected sensors can be modeled as factors adjusting the generate rate.

With the given weight factor $\omega_{i}$, service rate μ, total generation rate λ, and adjustable ${\left\{\lambda_{i}\right\}}_{i\in I}$ under the constraint $\lambda =\sum_{i=1}^{N}\lambda_{i}$, our goal was to minimize the weighted average AoI of the system by optimizing the generation rate of sources. Accordingly, for a multi-source system with self-preemption, the above problem can be formulated as Problem $P_{1}$, as shown in the following.

(2) $$\begin{array}{cc} P_{1}:\min_{\left\{\lambda_{i}:\;i\in I\right\}} & \;{\bar{\Delta }}_{\mathrm{self}} \end{array}$$

(2a) $$\begin{array}{cc} \;\;\;s.\;t.\;\; & \;\sum_{i=1}^{N}\lambda_{i}-\lambda =0, \end{array}$$

(2b) $$\begin{array}{cc} \;\;\;\;\;\;\; & \;\lambda_{i}>0,\forall i\in I, \end{array}$$

in which ${\bar{\Delta }}_{\mathrm{self}}$ is the weighted average AoI of the self-preemptive system.

Similarly, we have Problem $P_{2}$ for a multi-source system with global-preemption.

(3) $$\begin{array}{cc} P_{2}:\min_{\left\{\lambda_{i}:\;i\in I\right\}} & \;{\bar{\Delta }}_{\mathrm{glob}} \end{array}$$

(3a) $$\begin{array}{cc} \;\;\;s.\;t.\;\; & \;\sum_{i=1}^{N}\lambda_{i}-\lambda =0, \end{array}$$

(3b) $$\begin{array}{cc} \;\;\;\;\;\;\; & \;\lambda_{i}>0,\forall i\in I, \end{array}$$

in which ${\bar{\Delta }}_{\mathrm{glob}}$ is the weighted average AoI of the global-preemptive system.

For the multi-source M/M/1/1 systems with the above two different preemption strategies, we shall separately find the optimal allocation of the generation rate of sources to achieve the best overall timeliness performance.

It is expected that by optimizing the generation rate of each source, the overall timeliness of the system, e.g., the automatic driving system can be improved, and some significant insights can be obtained for system design.

## 4. Optimal Status Update Generation Rate under Self-Preemption Strategy and Global-Preemption Strategy

In this section, we first prove that the generation rate optimization problem is convex as shown in Proposition 1 and give the corresponding KKT conditions for the self-preemption system. To obtain the optimal generation rate under the self-preemption strategy, we present a basic convex optimization algorithm—Algorithm 1 as an example. Furthermore, we propose a set of closed-form approximate optimal generation rates with high accuracy as shown in Proposition 2. Additionally, to facilitate the further analysis and comparison with the overall freshness of the self-preemptive strategy, we also derive a set of upper bound and lower bound for the minimal weighted average AoI in Proposition 3. For the global-preemption strategy, we directly derived the corresponding optimal generation rate as shown in Proposition 4.

### 4.1. Optimal Generation Rate Allocation under Self-Preemption Strategy

In this subsection, we consider a multi-source M/M/1/1 system with self-preemption in which only updates from the same source can preempt each other. We optimize the generation rate of sources to achieve the optimal overall timeliness of the system under the generation rate control scheme.

As shown in [17], the average AoI of source *i* in the self-preemptive system is given by

(4) $$\begin{array}{c} \Delta_{i,\mathrm{self}}=\frac{\rho_{i}^{3}+\rho_{i}^{2}\left(2\rho_{-i}+3\right)+\rho_{i}\left(\rho_{-i}^{2}+5\rho_{-i}+3\right)+{\left(\rho_{-i}+1\right)}^{2}}{\mu \rho_{i}\left(\rho_{i}+1\right)\left(\rho +1\right)}, \end{array}$$

where $\rho_{-i}:=\sum_{j\in I\setminus \left\{i\right\}}\rho_{j}$ and $\rho_{i}+\rho_{-i}=\rho$.

By substituting (4) into (1), the weighted average AoI of the self-preemptive system is given by

(5) $$\begin{array}{c} {\bar{\Delta }}_{\mathrm{self}}=\sum_{i=1}^{N}\omega_{i}\left(\frac{\rho_{i}^{3}+\rho_{i}^{2}\left(2\rho_{-i}+3\right)+\rho_{i}\left(\rho_{-i}^{2}+5\rho_{-i}+3\right)+{\left(\rho_{-i}+1\right)}^{2}}{\mu \rho_{i}\left(\rho_{i}+1\right)\left(\rho +1\right)}\right). \end{array}$$

To optimize the generation rate of sources, we first analyze the convexity of $P_{1}$.

**Proposition** **1.** *$P_{1}$ is a convex problem.*

**Proof.**  By using expression $\rho_{i}=\rho -\rho_{-i}$ and performing some simplifications, (5) can be rewritten as

(6) $$\begin{array}{cc} {\bar{\Delta }}_{\mathrm{self}} & =\sum_{i=1}^{N}\frac{\omega_{i}}{\mu \left(1+\rho \right)}\left(\frac{1+\rho }{1+\rho_{i}}+\frac{{\left(1+\rho \right)}^{2}}{\rho_{i}}-1\right)\\ \; & =-\frac{1}{\mu \left(1+\rho \right)}+\sum_{i=1}^{N}\frac{\omega_{i}}{\mu }\left(\frac{1}{1+\rho_{i}}+\frac{1+\rho }{\rho_{i}}\right)\\ & =-\frac{1}{\mu +\lambda }+\sum_{i=1}^{N}\omega_{i}\left(\frac{1}{\lambda_{i}+\mu }+\frac{1+\rho }{\lambda_{i}}\right) \end{array}$$

Therefore, the first and second partial derivatives of ${\bar{\Delta }}_{\mathrm{self}}$ w. r. t. $\lambda_{i}$ are

(7) $$\begin{array}{c} \frac{\partial {\bar{\Delta }}_{\mathrm{self}}}{\partial \lambda_{i}}=\omega_{i}\left(-\frac{1}{{\left(\lambda_{i}+\mu \right)}^{2}}-\frac{1+\rho }{{\lambda_{i}}^{2}}\right) \end{array}$$

and

(8) $$\begin{array}{c} \frac{\partial^{2}{\bar{\Delta }}_{\mathrm{self}}}{\partial {\lambda_{i}}^{2}}=2\omega_{i}\left(\frac{1}{{\left(\lambda_{i}+\mu \right)}^{3}}+\frac{1+\rho }{{\lambda_{i}}^{3}}\right)>0, \end{array}$$

(9) $$\begin{array}{c} \frac{\partial^{2}{\bar{\Delta }}_{\mathrm{self}}}{\partial \lambda_{i}\partial \lambda_{j}}=0,\;\;\forall i\in I,j\in I\setminus \left\{i\right\}. \end{array}$$

By checking the positive definitiness of the Hessian matrix, one can prove the convexity of ${\bar{\Delta }}_{\mathrm{self}}$ w. r. t. $\left(\lambda_{1},\lambda_{2},\cdots ,\lambda_{N}\right)$. To be specific, the Hessian matrix of ${\bar{\Delta }}_{self}$ is given by

(10) $$H_{{\bar{\Delta }}_{\mathrm{self}}}=\left[\begin{array}{cccc} \frac{\partial^{2}{\bar{\Delta }}_{\mathrm{self}}}{\partial {\lambda_{1}}^{2}} & \frac{\partial^{2}{\bar{\Delta }}_{\mathrm{self}}}{\partial \lambda_{1}\partial \lambda_{2}} & \cdots & \frac{\partial^{2}{\bar{\Delta }}_{\mathrm{self}}}{\partial \lambda_{1}\partial \lambda_{N}}\\ \frac{\partial^{2}{\bar{\Delta }}_{\mathrm{self}}}{\partial \lambda_{2}\partial \lambda_{1}} & \frac{\partial^{2}{\bar{\Delta }}_{\mathrm{self}}}{\partial {\lambda_{2}}^{2}} & \cdots & \frac{\partial^{2}{\bar{\Delta }}_{\mathrm{self}}}{\partial \lambda_{2}\partial \lambda_{N}}\\ \vdots & \vdots & \ddots & \vdots \\ \frac{\partial^{2}{\bar{\Delta }}_{\mathrm{self}}}{\partial \lambda_{N}\partial \lambda_{1}} & \frac{\partial^{2}{\bar{\Delta }}_{\mathrm{self}}}{\partial \lambda_{N}\partial \lambda_{2}} & \cdots & \frac{\partial^{2}{\bar{\Delta }}_{\mathrm{self}}}{\partial {\lambda_{N}}^{2}} \end{array}\right].$$

From (8) and (9), one can notice that the Hessian matrix is a diagonal matrix, in which elements on diagonal are positive. Hence, the eigenvalues of the Hessian matrix are positive, which implies that the Hessian matrix of ${\bar{\Delta }}_{\mathrm{self}}$ is positive definite. Taking into account the fact that the feasible set of $P_{1}$ is convex, it is concluded that ${\bar{\Delta }}_{\mathrm{self}}$ is a convex function w. r. t. $\left(\lambda_{1},\lambda_{2},\cdots ,\lambda_{N}\right)$ under the condition of $0<\lambda_{i}<\lambda$, which contains the feasible set. □

Then, we write the corresponding Lagrangian function as follows:

(11) $$\begin{array}{c} L={\bar{\Delta }}_{\mathrm{self}}+\theta \left(\sum_{i=1}^{N}\lambda_{i}-\lambda \right)-\sum_{j=1}^{N}\gamma_{i}\lambda_{i}, \end{array}$$

in which θ is a real number and $\gamma_{i}\geq 0$ for ∀i∈I. Since $P_{1}$ is a convex problem, the KKT conditions are sufficient and necessary conditions for the optimal solution, which are

(12) $$\begin{array}{c} \frac{\partial L}{\partial \lambda_{i}}=-\omega_{i}\left(\frac{1}{{\left(\lambda_{i}+\mu \right)}^{2}}+\frac{1+\rho }{{\lambda_{i}}^{2}}\right)+\theta -\gamma_{i}=0, \end{array}$$

(13) $$\begin{array}{c} \sum_{i=1}^{N}\lambda_{i}-\lambda =0, \end{array}$$

(14) $$\begin{array}{c} \lambda_{i}>0, \end{array}$$

(15) $$\begin{array}{c} \gamma_{i}\geq 0, \end{array}$$

(16) $$\begin{array}{c} \gamma_{i}\lambda_{i}=0, \end{array}$$

for ∀i∈I. Moreover, from the conditions (14)–(16), the KKT conditions can be simplified as

(17) $$\begin{array}{c} \theta -\omega_{i}\left(\frac{1}{{\left(\lambda_{i}+\mu \right)}^{2}}+\frac{1+\rho }{{\lambda_{i}}^{2}}\right)=0, \end{array}$$

(18) $$\begin{array}{c} \sum_{i=1}^{N}\lambda_{i}-\lambda =0, \end{array}$$

(19) $$\begin{array}{c} \lambda_{i}>0, \end{array}$$

for ∀i∈I.

Due to the complexity of (17)–(19), we cannot obtain the optimal solution by solving them directly. As an alternative, we can use convex optimization algorithms to obtain the optimal solution since we have already established the convexity of $P_{1}$. To be specific, let us give a basic convex optimization algorithm as an example.

We consider using Newton’s method combined with the penalty function method by adding a barrier function. Before presenting the algorithm, we first denote the following notations: $\Lambda ={\left[\lambda_{1},\lambda_{2},\cdots \lambda_{N}\right]}^{T}$, $A={\left[1,1,\cdots ,1\right]}_{N\times 1}$ and

(20) $$\begin{array}{c} G\left(\Lambda \right)={\bar{\Delta }}_{\mathrm{self}}\left(\Lambda \right)-\sum_{i=1}^{N}k_{i}\ln\lambda_{i}, \end{array}$$

where $k_{i}$$\left(0<k_{i}<1\right)$ is reduction coefficient of the penalty factor. Based on Newton’s method, we can obtain the increment δΛ of Λ in each iteration by solving the matrix equation

(21) $$\left(\begin{array}{cc} \nabla^{2}G\left(\Lambda \right) & A^{T}\\ A & 0 \end{array}\right)\left(\begin{array}{c} \delta \Lambda \\ W \end{array}\right)=\left(\begin{array}{c} -\nabla G\left(\Lambda \right)\\ 0 \end{array}\right).$$

By considering the fact that $H_{{\bar{\Delta }}_{\mathrm{self}}}$ is positive definite, one can check that $\nabla^{2}G\left(\Lambda \right)$ is also positive definite, which implies that the KKT matrix is a non-singular matrix. Therefore, it can be deduced that Equation (21) in the algorithm always has a solution. This provides the possibility to compute the Newton decrement *d* via

(22) $$d={\left(\delta \Lambda^{T}\nabla^{2}G\left(\Lambda \right)\delta \Lambda \right)}^{1/2}.$$

With these preparations, let us present the algorithm as shown in Algorithm 1. Particularly, the contribution and the reason for proposing Algorithm 1 are stated as follows.

- *Contribution of Algorithm 1:* A basic and typical tool for solving the optimal generation rates under the self-preemption strategy.
- *Reason of proposing Algorithm 1:* According to the complexity of the obtained KKT conditions, we find that it is difficult to solve the optimal solution directly, and, for a convex problem, there are a large number of mature convex optimization algorithms that can be used to solve the optimal solution. It is for these reasons that we proposed a typical and basic algorithm, Algorithm 1, as an easy-to-use tool and example.

**Algorithm 1: **Newton- ${\bar{\Delta }}_{\mathrm{self}}$ Minimizer.  **Input**: $\omega_{i}>0$, $\mu_{i}>0$, λ>0, reduction coefficient of the penalty factor $0<k_{i}<1$, i∈I, convergence range ε and number of iterations *M*. 
  **Output**: Optimal generation rate $\Lambda^{🟉}$
**1**   Initialize the iterations counter to j=1, Newton decrement d≫ε andgeneration rate $\Lambda ={\left[1/N,\cdots ,1/N\right]}^{T}$.**2**   **while** *j≤M* **do**(
**3**       Compute increment δΛ and *W* via matrix equation (21);**4**       Update Newton decrement *d* via Equation (22);**5**       **if** $\frac{d^{2}}{2}>\varepsilon$ **then**(
**6**         Choose step size *l* by backtracking line search;**7**         Update candidate optimal generation rate Λ=Λ+lδΛ;**8**         Update iteration counter j=j+1;**9**       **else**(**10**         break;**11**     **end****12**   (**end**(**13**   Return Optimal generation rate $\Lambda^{🟉}=\Lambda$

In some scenarios, the computing resources for the system are limited to completing the convex optimization algorithms. Hence, a closed-form approximate generation rate would instead be used by the system to save the computing resources and efficiently allocate the total generation rate. In systems with high data collection costs, if the status update is too frequent, the overall system overhead would be very high. For instance, the update frequency in the ecological environment monitoring system should be controlled for prolonging the equipment battery life, especially in remote areas. In this case, smaller generation rates are required, and the system works under a light load. Based on these observations, we investigate the approximate generation rate of the self-preemptive system with light load where the total load ρ→0, as shown in Proposition 2. Let us first prove the following lemma.

**Lemma** **1.** *Suppose $x_{i}>0$, $\;y_{i}>0$, ∀i∈I. Inequality $\sum_{i=1}^{N}\frac{x_{i}}{y_{i}}\geq {\left(\sum_{i=1}^{N}\sqrt{x_{i}}\right)}^{2}/\sum_{i=1}^{N}y_{i}$ holds, and the only condition that the equal sign holds is $y_{i}\sqrt{x_{j}}=y_{j}\sqrt{x_{i}}$, ∀i,j∈I.*

**Proof.**  See Appendix A. □

Based on Lemma 1, the approximate optimal generation rates to $P_{1}$ can be readily derived in the following proposition.

**Proposition** **2.** *The optimal generation rates of the self-preemption system in the light load case can be approximated as*

(23) $$\begin{array}{c} \lambda_{i}=\frac{\sqrt{\omega_{i}}}{\sum_{i=1}^{N}\sqrt{\omega_{i}}}\lambda ,\;\;\forall i\in I. \end{array}$$

**Proof.**  Since ρ→0 implies that $\rho_{i}\to 0$, (6) can, therefore, be approximated as

(24) $$\begin{array}{cc} {\bar{\Delta }}_{\mathrm{self}} & \approx -\frac{1}{\mu \left(1+\rho \right)}+\sum_{i=1}^{N}\frac{\omega_{i}}{\mu }\left(1+\frac{1+\rho }{\rho_{i}}\right)\\ \; & =\frac{\rho }{\mu \left(1+\rho \right)}+\frac{1+\rho }{\mu }\sum_{i=1}^{N}\frac{\omega_{i}}{\rho_{i}}\\ \; & \overset{\left(a\right)}{\geq }\frac{\rho }{\mu \left(1+\rho \right)}+\frac{1+\rho }{\mu }{\left(\sum_{i=1}^{N}\sqrt{\omega_{i}}\right)}^{2}/\sum_{i=1}^{N}\rho_{i}\\ \; & =\frac{\rho }{\mu \left(1+\rho \right)}+\frac{\left(1+\rho \right)}{\lambda }{\left(\sum_{i=1}^{N}\sqrt{\omega_{i}}\right)}^{2}, \end{array}$$

in which $\left(a\right)$ follows from Lemma 1, and the only condition that the equal sign holds is $\lambda_{i}\sqrt{\omega_{j}}=\lambda_{j}\sqrt{\omega_{i}}$, ∀i,j∈I. Thus, one can set that

(25) $$\begin{array}{c} \frac{\lambda_{i}}{\sqrt{\omega_{i}}}=t,\forall i\in I. \end{array}$$

After processing the equation, we obtain

(26) $$\begin{array}{c} \lambda =\sum_{i=1}^{N}\lambda_{i}=t\sum_{i=1}^{N}\sqrt{\omega_{i}}, \end{array}$$

which implies that $t=\lambda /\sum_{i=1}^{N}\sqrt{\omega_{i}}$. By combining (25), we find that the equal sign of (a) holds if and only if $\lambda_{i}=\sqrt{\omega_{i}}\lambda /\sum_{i=1}^{N}\sqrt{\omega_{i}},\forall i\in I$.

According to obtained results, the error brought by the approximation is minimal and can be ignored. Therefore, (23) can be applied to approximate the optimal generation rate in the light load case, which minimizes the weighted average AoI of the self-preemptive system. □

Note that the results in Proposition 2 for the light load case can also be applicable to other load cases, which will be verified later in Section 5. Particularly, it can also be seen that the optimal generation rate of each source is equal when the timeliness requirements of each source are the same, i.e., λi=λλnN,∀i∈I, which means the approximate optimal generation rate proposed in Proposition 2 is the same as the optimal generation rate in this case.

Additionally, to facilitate further analysis and comparison with the overall freshness of the self-preemptive strategy, we also found a set of upper bounds ${\bar{\Delta }}_{\min,\mathrm{self}}^{\mathrm{ub}}$ and lower bounds ${\bar{\Delta }}_{\min,\mathrm{self}}^{\mathrm{lb}}$ of the minimal weighted average AoI ${\bar{\Delta }}_{\min,\mathrm{self}}$, as shown in the following proposition.

**Proposition** **3.** *A set of lower bound and upper bound of the minimal weighted average AoI is derived as*

(27) $$\begin{array}{c} {\bar{\Delta }}_{\min,\mathrm{self}}^{\mathrm{lb}}=\frac{1+\rho }{\lambda }{\left(\sum_{i=1}^{N}\sqrt{\omega_{i}}\right)}^{2}, \end{array}$$

*and*

(28) Δ¯min,selfub=−1μ+λ+1+ρ∑i=1Nωi21+ρ∑i=1Nωi2λλ+∑i=1Nωiωiμ+λωiλωi∑i=1Nωi∑i=1Nωiμ+λωiλωi∑i=1Nωi∑i=1Nωi.

**Proof.**  Based on (6) and the condition $\rho \geq \rho_{i}\geq 0$,

(29) $$\begin{array}{c} {\bar{\Delta }}_{\mathrm{self}}\geq \left(1+\rho \right)\sum_{i=1}^{N}\frac{\omega_{i}}{\lambda_{i}}. \end{array}$$

Note that (29) holds for $\forall \left(\lambda_{1},\lambda_{2},\cdots ,\lambda_{N}\right)$. Therefore, we have

(30) $$\begin{array}{cc} {\bar{\Delta }}_{\min,\mathrm{self}} & \geq \min\left\{\left.\left(1+\rho \right)\sum_{i=1}^{N}\frac{\omega_{i}}{\lambda_{i}}\right\}\right.\\ \; & \overset{\left(b\right)}{=}\frac{\left(1+\rho \right)}{\lambda }{\left(\sum_{i=1}^{N}\sqrt{\omega_{i}}\right)}^{2}, \end{array}$$

where $\left(b\right)$ follows from Lemma 1. We, therefore, obtain a lower bound of ${\bar{\Delta }}_{\min,\mathrm{self}}$.

By substituting the results on Proposition 2 into (6), an approximate minimal weighted average AoI can be derived, which can also be an upper bound of ${\bar{\Delta }}_{\min,\mathrm{self}}$. Therefore, (28) is obtained. This ends the proof. □

### 4.2. Optimal Generation Rate of Different Sources under Global-Preemption Strategy

In this subsection, we study a multi-source M/M/1/1 system with global-preemption. We optimize the generation rate of sources to achieve the optimal overall timeliness of the system under the generation rate control scheme.

The multi-source M/M/1/1 system with global-preemption was comprehensively studied in [16]. Accordingly, the average AoI of source *i* is

(31) $$\begin{array}{c} \Delta_{i,\mathrm{glob}}=\frac{1}{\lambda_{i}E\left(e^{-\lambda S}\right)}, \end{array}$$

where *E* represents the expectation operator and *S* is the service time that follows negative exponential distribution with parameter μ. Thus,

(32) $$\begin{array}{c} E\left(e^{-\lambda S}\right)=\int_{0}^{+\infty }\mu e^{-\left(\lambda +\mu \right)s}ds=\frac{\mu }{\lambda +\mu }. \end{array}$$

Therefore, the weighted average AoI of the global-preemptive system is given by

(33) $$\begin{array}{c} {\bar{\Delta }}_{\mathrm{glob}}=\frac{\mu +\lambda }{\mu }\sum_{i=1}^{n}\frac{\omega_{i}}{\lambda_{i}}. \end{array}$$

Recalling (3), it is obvious that $P_{2}$ is a convex problem. However, unlike the analysis for the self-preemptive system, the true optimal generation rates for $P_{2}$ can be derived directly by using Lemma 1, as shown in the following proposition.

**Proposition** **4.** *The optimal generation rates of the global-preemption system can be derived as*

(34) $$\begin{array}{c} \lambda_{i}=\frac{\sqrt{\omega_{i}}}{\sum_{i=1}^{N}\sqrt{\omega_{i}}}\lambda ,\;\;\forall i\in I. \end{array}$$

**Proof.**  By using Lemma 1,

(35) $$\begin{array}{cc} {\bar{\Delta }}_{\mathrm{glob}} & =\frac{\mu +\lambda }{\mu }\sum_{i=1}^{n}\frac{\omega_{i}}{\lambda_{i}}\\ \; & \overset{\left(c\right)}{\geq }\frac{\mu +\lambda }{\mu }{\left(\sum_{i=1}^{N}\sqrt{\omega_{i}}\right)}^{2}/\sum_{i=1}^{N}\lambda_{i}\\ \; & ={\left(\sum_{i=1}^{N}\sqrt{\omega_{i}}\right)}^{2}\left(\frac{1}{\mu }+\frac{1}{\lambda }\right). \end{array}$$

in which $\left(c\right)$ follows from Lemma 1, and the only condition that the equal sign holds is $\lambda_{i}\sqrt{\omega_{j}}=\lambda_{j}\sqrt{\omega_{i}}$, ∀i,j∈I. With the same manipulations in (25) and (26), we find that $\lambda_{i}=\sqrt{\omega_{i}}\lambda /\sum_{i=1}^{N}\sqrt{\omega_{i}},\forall i\in I$. This ends the proof. □

From (23) and (34), we surprisingly find that the optimal generation rates under the global-preemption strategy are the same as the approximate optimal generation rates under the self-preemption strategy.

Additionally, from (30) and (35), we find that the lower bound of minimal weighted average AoI of the self-preemption strategy is the same as that of the global-preemption strategy, which indicates that the performance of the global-preemption system is better than that of the self-preemption system in terms of the overall timeliness.

## 5. Numerical Analysis of the Weighed Average AoIs of Preemptive Systems

In this section, we first provide numerical analysis for the weighed average AoIs of the self-preemptive system and the global-preemptive system, i.e, ${\bar{\Delta }}_{\mathrm{self}}$ and ${\bar{\Delta }}_{\mathrm{glob}}$, to show the optimization brought by generation rate control. Then, for the system with self-preemption, the given numerical examples illustrate the accuracy of the approximate optimal generation rate. Finally, we compare the performance of the self-preemptive system and the global-preemptive system. In all of the numerical results, we assume N=2 and μ=1.

**Figure 2:**
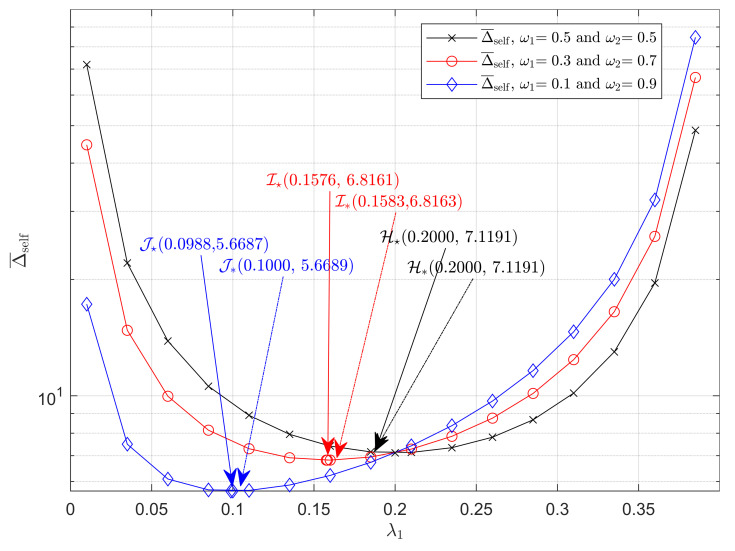
${\bar{\Delta }}_{\mathrm{self}}$ as a function of $\lambda_{1}$ under self-preemption strategy. μ=1, λ=0.4. $\left(\omega_{1},\omega_{2}\right)$ takes values (0.5,0.5), (0.3,0.7), and (0.1,0.9).

Figure 2 shows the weighted average AoI under the self-preemptive strategy, i.e., ${\bar{\Delta }}_{\mathrm{self}}$, for different values of $\lambda_{1}$. By substituting the settings μ=1 and λ=0.4 into (5), we obtain three curves of ${\bar{\Delta }}_{\mathrm{self}}$, which respectively corresponds to $\omega_{1}=0.5$, $\omega_{1}=0.3$ and $\omega_{1}=0.1$. First, we observed that ${\bar{\Delta }}_{\mathrm{self}}$ decreases first and then increases as the generation rate of source 1 $\lambda_{1}$ increases for all the three curves. Moreover, it can be seen from the above curves that ${\bar{\Delta }}_{\mathrm{self}}$ is a convex function with respect to $\lambda_{1}$, which verifies Proposition 2.

Secondly, the points $H_{🟉}$, $I_{🟉}$, and $J_{🟉}$, which, respectively, represent the minimum of ${\bar{\Delta }}_{\mathrm{self}}$(obtained by using Algorithm 1 for different $\omega_{1}$) when $\omega_{1}$ is set to 0.5, 0.3, and 0.1 respectively, and points $H_{*}$, $I_{*}$, and $J_{*}$, which are the approximate minimums of ${\bar{\Delta }}_{\mathrm{self}}$(obtained by combining (5) and (23)), show that the overall timeliness of the system can be optimized by generation rate control. Furthermore, it can be seen that the optimal generation rate of source 1 $\lambda_{1}$ increases as the weight factor $\omega_{1}$ increases according to the points $H_{🟉}$, $I_{🟉}$ and $J_{🟉}$ because the more urgent source needs a higher generation rate. In fact, this can be also deduced from (23).

It is also important to note that the positions of $H_{🟉}$ and $H_{*}$, $I_{🟉}$ and $I_{*}$, and $J_{🟉}$ and $J_{*}$ are all very close, which implies that the proposed approximations on optimal generation rate has a high accuracy, and this will be analyzed further in Figure 4. Additionally, recalling the automatic driving system using the self-preemption strategy mentioned earlier, we set $\lambda_{1}$ to be the corresponding generation rate of engine temperature and $\lambda_{2}$ to be that of vehicle speed. One can find that by optimizing the generation rate, the overall timeliness of the system is improved.

**Figure 3:**
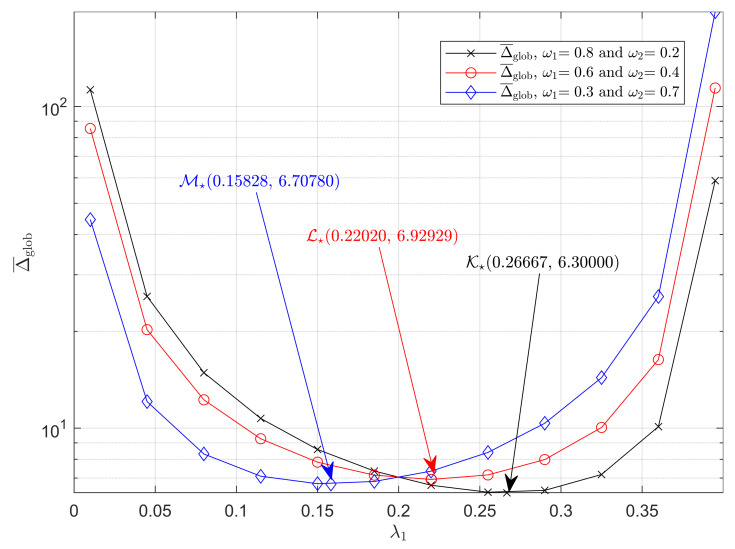
${\bar{\Delta }}_{\mathrm{glob}}$ as a function of $\lambda_{1}$ under global-preemption strategy. μ=1, λ=0.4. $\left(\omega_{1},\omega_{2}\right)$ takes values (0.8,0.2), (0.6,0.4), and (0.3,0.7) respectively.

Figure 3 shows the weighted average AoI under global-preemptive strategy, i.e., ${\bar{\Delta }}_{\mathrm{glob}}$, for different values of $\lambda_{1}$.Here, we similarly set μ=1 and λ=0.4 and depicts three curves of ${\bar{\Delta }}_{\mathrm{self}}$ based on (33), which, respectively, correspond to $\omega_{1}=0.8$, $\omega_{1}=0.6$, and $\omega_{1}=0.3$.Then, we obtain the minimums of ${\bar{\Delta }}_{\mathrm{glob}}$ for all the three curves according to (35). Points $K_{🟉}$, $L_{🟉}$ and $M_{🟉}$ are the minimums of ${\bar{\Delta }}_{\mathrm{glob}}$ correspond to the cases when $\omega_{1}=0.8$, $\omega_{1}=0.6$ and $\omega_{1}=0.3$, respectively. Similar to the analysis for Figure 2, one can verify that $P_{2}$ is a convex problem and check that the information freshness of the system can be improved by controlling the generation rate.

Additionally, it can also be deduced that a lower generation rate must be allocated to a less urgent source because the optimal generation rate of source 1 $\lambda_{1}$ decreases as the weight factor $\omega_{1}$ decreases. Recalling the automatic driving system using the global-preemption strategy mentioned earlier, for the black and red curves, we set $\lambda_{1}$ to be the corresponding generation rate of vehicle speed and $\lambda_{2}$ to be that of the engine temperature. It can be seen that, by optimizing the generation rate, the overall timeliness of this system is improved.

To evaluate the accuracy of the approximate optimal generation rates proposed for the self-preemption strategy, we first introduce the metric η, which measures the relative error between approximation and true optimum.

(36) $$\begin{array}{c} \eta :=\frac{{\bar{\Delta }}_{\mathrm{self}}^{*}-{\bar{\Delta }}_{\mathrm{self}}^{🟉}}{{\bar{\Delta }}_{\mathrm{self}}^{*}}, \end{array}$$

in which ${\bar{\Delta }}_{\mathrm{self}}^{🟉}$ is the weighted average AoI when the generation rate of sources is set following the true optimum and ${\bar{\Delta }}_{\mathrm{self}}^{*}$ is that achieved by using the approximate optimal generation rate, i.e., ${\bar{\Delta }}_{\mathrm{self}}^{*}={\bar{\Delta }}_{\mathrm{self}}\left(\frac{\sqrt{\omega_{1}}}{\sqrt{\omega_{1}}+\sqrt{\omega_{2}}}\lambda ,\frac{\sqrt{\omega_{2}}}{\sqrt{\omega_{1}}+\sqrt{\omega_{2}}}\lambda \right)$. The smaller η is, the higher accuracy the approximate optimal generation rate can achieve.

**Figure 4:**
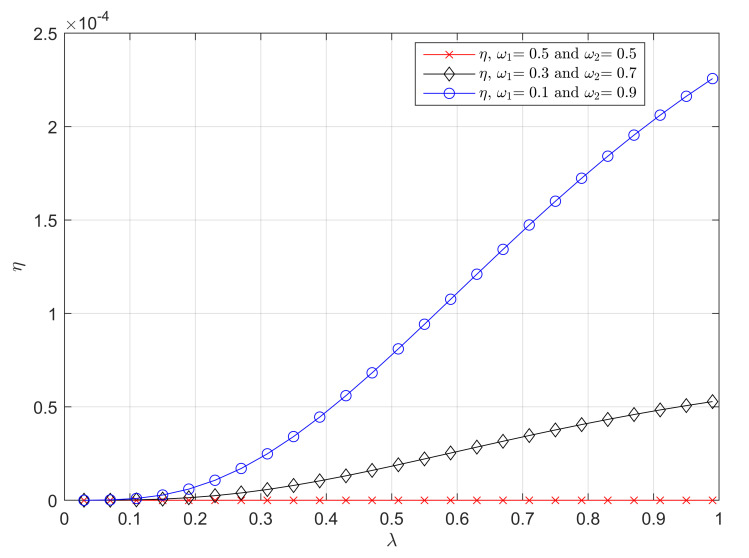
η as a function of λ. μ=1. $\left(\omega_{1},\omega_{2}\right)$ takes values (0.5,0.5), (0.3,0.7), and (0.1,0.9).

In Figure 4, we investigate how the relative error η varies with the total generation rate λ. The weight factor $\omega_{1}$ is set to 0.5, 0.3 and 0.1. We first use Algorithm 1 to obtain the minimums of ${\bar{\Delta }}_{\mathrm{self}}$ under different $\omega_{1}$, i.e., $\omega_{1}$=0.5, 0.3, 0.1 and λ. According to (5), (23) and the obtained minimums of ${\bar{\Delta }}_{\mathrm{self}}$, we calculate the relative error η based on (36) and depict three curves of η, which correspond to $\omega_{1}=0.5$, $\omega_{1}=0.3$, and $\omega_{1}=0.1$. One can see that relative error η is extremely small when λ<0.2 (ρ<0.2) for every $\omega_{1}$, which verifies that the approximate optimal generation rate under a light load (Proposition 2) is highly accurate.

It can also be seen in Figure 4 that, when $\omega_{1}$ takes general values($\omega_{1}\neq 0.5$), the relative error η increases as λ, i.e., ρ increases. This is because the approximate optimal generation rates were originally proposed for light load cases. However, η is still relatively small ($\eta <2.5\times 10^{-4}$) in Figure 4 for all λ∈(0,1), which means that the approximate optimal generation rate can achieve a high accuracy under any system load and can be applied to any load. Particularly, η always equals to 0 when $\omega_{1}=0.5$, i.e., η=0, this is because the approximate optimal generation rate is equivalent to the optimal generation rate when the urgency requirements for each source are the same.

For instance, for the automatic driving system that adopts a self-preemption strategy, we use the same settings as the analysis in Figure 2. The accuracy of the proposed approximate optimal generation rate is excellent, which means that this set of approximate optimal generation rates has extremely high application value in the case of limited terminal computing resources or rapid deployment.

**Figure 5:**
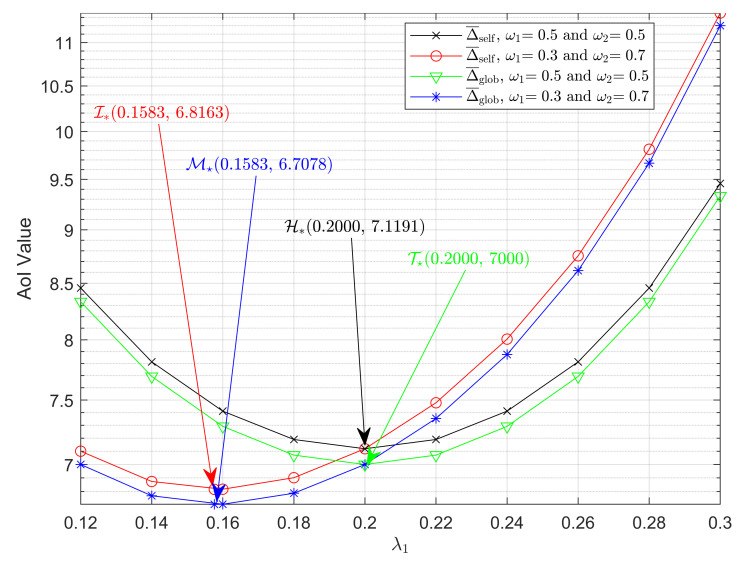
${\bar{\Delta }}_{\mathrm{self}}$ and ${\bar{\Delta }}_{\mathrm{glob}}$ as functions of λ. μ=1, λ=0.4. $\left(\omega_{1},\omega_{2}\right)$ takes values (0.5,0.5) and (0.3,0.7).

In Figure 5, we compare the weighted average AoIs of the self-preemptive system and the global-preemptive system. λ=0.4, and the weight factor $\omega_{1}$ is set to 0.5 and 0.3, respectively. By using (5), we first obtain the black curve when $\omega_{1}=0.5$ and the red curve $\omega_{1}=0.3$, which show how the weighted average AoI varies with the generation rate $\lambda_{1}$ under self-preemption. Likewise, for the global-preemption, the green curve when $\omega_{1}=0.5$ and the blue curve when $\omega_{1}=0.3$ can also be obtained by using (33). According to (5) and (23), we find the approximate minimums of ${\bar{\Delta }}_{\mathrm{self}}$ when $\omega_{1}$ takes 0.5 and 0.3. Correspondingly, the minimums of ${\bar{\Delta }}_{\mathrm{glob}}$ for different $\omega_{1}$ can be found according to (35).

Point $T_{🟉}$ is the minimum of ${\bar{\Delta }}_{\mathrm{glob}}$ in the case when $\omega_{1}=0.5$, and the points $H_{🟉}$, $I_{🟉}$, and $M_{🟉}$ were defined earlier. It can be seen that, under the same weight setting, ${\bar{\Delta }}_{\mathrm{glob}}$ is always smaller than ${\bar{\Delta }}_{\mathrm{self}}$, which is consistent with the conclusion in [17]. Moreover, we notice that the abscissas of points $I_{🟉}$ and $M_{🟉}$ and points $H_{🟉}$ and $T_{🟉}$ are the same, which is because the optimal generation rates under the global-preemption strategy are the same as the approximate optimal generation rates under the self-preemption strategy.

Additionally, from the curves and expressions (23) and (34), the overall timeliness of the global-preemptive system is very similar to that of the self-preemptive system. For the aforementioned automatic driving system, we set $\lambda_{1}$ to be the corresponding generation rate of engine temperature and $\lambda_{2}$ to be that of vehicle speed. According to Figure 5 and the previous analysis, one can find that, in terms of overall timeliness, the global-preemption strategy was more suitable for this system.

## 6. Conclusions

In this paper, we studied the overall timeliness of a multi-source sensing system with preemption. To optimize the overall timeliness performance of the preemptive system, we controlled the generation rate of sources according to the different urgency requirements for different sources. Specifically, we considered two preemption strategies, a self-preemption strategy and global-preemption strategy.

For the self-preemption strategy, the optimization problem was proven to be a convex problem by proving the positive semi-definiteness of the Hessian matrix of the objective function, and the optimal generation rate was obtained directly by using the convex optimization algorithm. We provided Algorithm 1 as a basic exemplary algorithm for reference. To meet the possible rapid deployment requirements in practice, a closed-form expression for an approximate optimal generation rate under the self-preemption strategy and light load was derived by using Lemma 1, which was based on Cauchy’s inequality.

After that, in order to gain more insights, we used Lemma 1 to derive a set of upper and lower bounds of the minimal weighted average AoI. For the global-preemption strategy, we also used Lemma 1 to derive a closed-form expression for the optimal generation rate. Through comparison, we found that the minimal weighted average AoI under the global-preemption strategy was exactly the same as the proposed lower bound of the minimal weighted average AoI under the self-preemption strategy, which indicates that the global-preemption strategy is better than the self-preemption strategy in terms of overall timeliness.

Numerical analysis validated our theoretical analyses and illustrated that: (1) The overall timeliness performance of the preemptive system was significantly improved by controlling generation rate. (2) The proposed approximate optimal generation rates for the self-preemption strategy were surprisingly accurate in the light load case and even in other cases. It could be seen in Figure 4 that the relative error η was no more than $2.5\times 10^{-4}$ for all total arrival rates λ∈(0,1).

## Appendix A. Proof of Lemma 1

Since $x_{i}>0$ and $\;y_{i}>0$ hold, ∀i∈I, one can find

(A1) $$\begin{array}{cc} \left(\sum_{i=1}^{N}\frac{x_{i}}{y_{i}}\right)\left(\sum_{i=1}^{N}y_{i}\right) & \overset{\left(d\right)}{\geq }{\left(\sum_{i=1}^{N}\sqrt{\frac{x_{i}}{y_{i}}}\cdot \sqrt{y_{i}}\right)}^{2}={\left(\sum_{i=1}^{N}\sqrt{x_{i}}\right)}^{2}, \end{array}$$

in which (d) follows from Cauchy’s inequality. Thus, one can find

(A2) $$\begin{array}{c} \sum_{i=1}^{N}\frac{x_{i}}{y_{i}}\geq {\left(\sum_{i=1}^{N}\sqrt{x_{i}}\right)}^{2}/\sum_{i=1}^{N}y_{i}, \end{array}$$

in which the equal sign holds if and only if $y_{i}\sqrt{x_{j}}=y_{j}\sqrt{x_{i}}$, ∀i,j∈I.
